# Supplementary material for: Electrochemical nitrate reduction in acid enables high-efficiency ammonia synthesis and high-voltage pollutes-based fuel cells
Source: Nat Commun. 2023 Dec 5;14:8036. doi: 10.1038/s41467-023-43897-6 (PMC10698138; doi:10.1038/s41467-023-43897-6)
Supplement: Supplementary file 1 — Supplementary Information [file 41467_2023_43897_MOESM1_ESM.pdf]

Supporting Information

Electrochemical Nitrate Reduction in Acid Enables High-Efficiency Ammonia Synthesis and High-Voltage Pollutes-Based Fuel Cells

Rong Zhang,[1] Chuan Li,[1] Huilin Cui,[1] Yanbo Wang,[1] Shaoce Zhang,[1] Pei Li,[1] Yue Hou,[1] Ying Guo,[2*] Guojin Liang,[1] Zhaodong Huang,[1] Chao Peng,[3,*] Chunyi Zhi[1,4,5*]

[1]Department of Materials Science and Engineering, City University of Hong Kong, 83 Tat Chee Avenue, 999077, Kowloon, Hong Kong, China

[2]College of Materials Science and Engineering, Shenzhen University, 518061, Shenzhen, China

[3]Multiscale Crystal Materials Research Center, Shenzhen Institute of Advanced Technology, Chinese Academy of Sciences, 518055, Shenzhen, China

[4]Centre for Functional Photonics, City University of Hong Kong, 999077, Kowloon, Hong Kong, China

[5]Songshan Lake Materials Laboratory, 523808, Dongguan, Guangdong, China

Corresponding author: Prof. Chunyi Zhi

Email: cy.zhi@cityu.edu.hk; yingguo@szu.edu.cn; chao.peng@siat.ac.cn

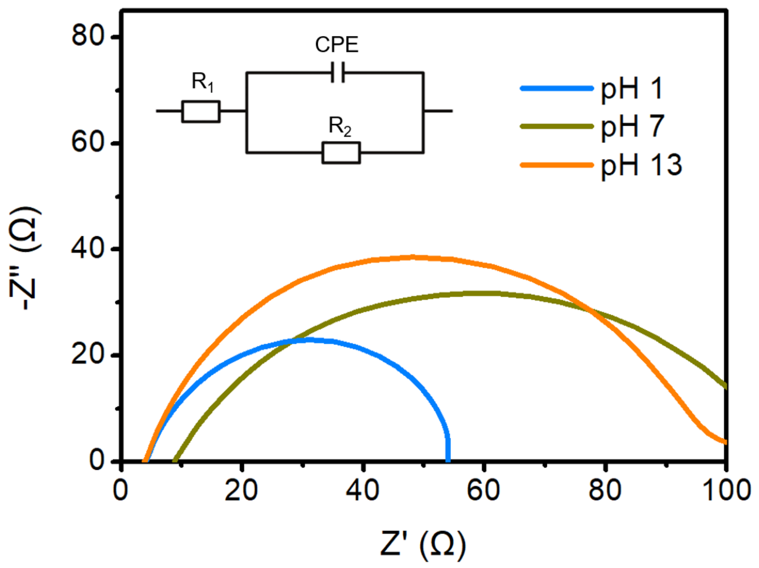

Supplementary Fig. 1. Nyquist plots for TiO_2 at different pH values. R_1 and R_2 is the resistances related to the potential loss and charge transfer at the electrode/electrolyte interface. CPE refers to the constant phase element. The fitting results for resistance values with error bars are shown in Supplementary Table 1. The R1 for TiO_2 at pH 1, 7 and 13 are determined as 4.51 ± 0.57 Ω, 8.86 ± 0.18 Ω and 3.94 ± 0.10 Ω, respectively. The R2 for TiO_2 at pH 1, 7 and 13 are determined as 52.28 ± 1.06 Ω, 100.10 ± 0.31 Ω and 95.04 ± 1.84 Ω, respectively.

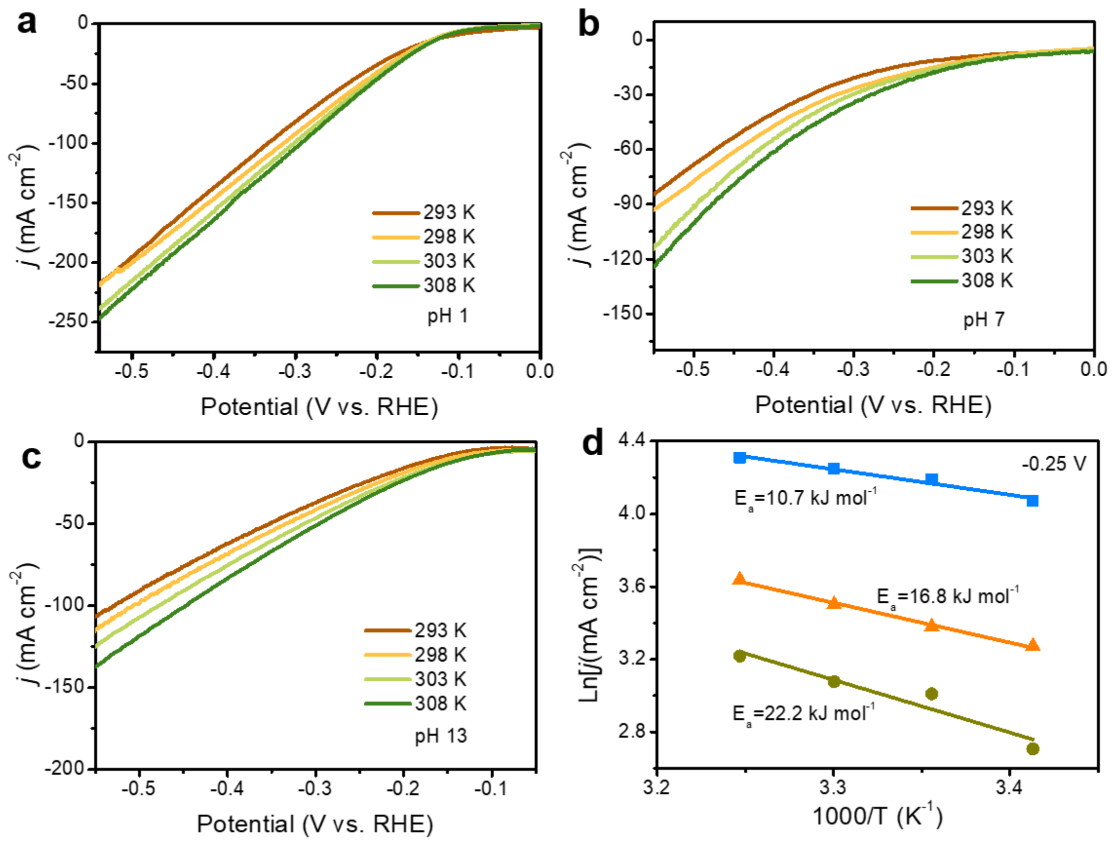

Supplementary Fig. 2. Calculation of E_a for nitrate reduction. LSV curves of the TiO_2 catalysts at different temperatures in the electrolyte containing 0.5 M NO_3^- with (a) pH 1, (b) pH 7 and (c) pH 13. (d) An Arrhenius plot showing the linear relationship between logarithmic values of the reciprocal of the catalytic current densities and the reciprocal of absolute temperatures for NO_3^-RR on the TiO_2 in 0.5 M NO_3^- electrolyte at −0.25 V.

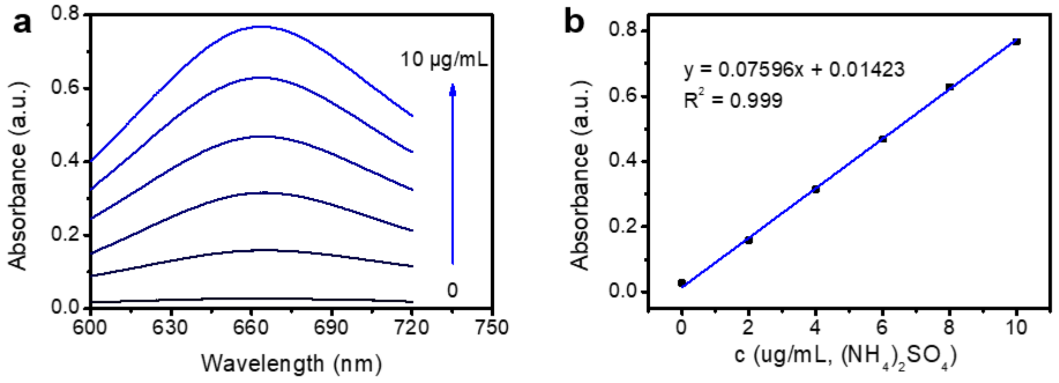

Supplementary Fig. 3. (a) UV-vis adsorption spectra and (b) standard curves for ammonia at pH1.

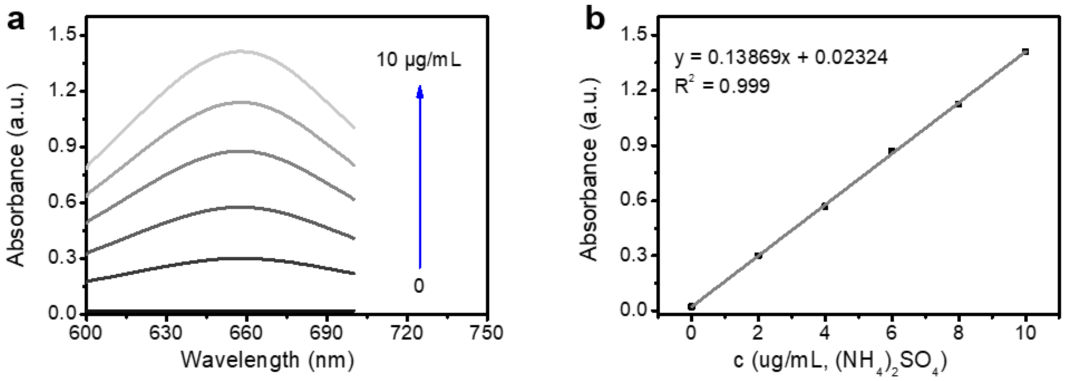

Supplementary Fig. 4. (a) UV-vis adsorption spectra and (b) standard curves for ammonia at pH7.

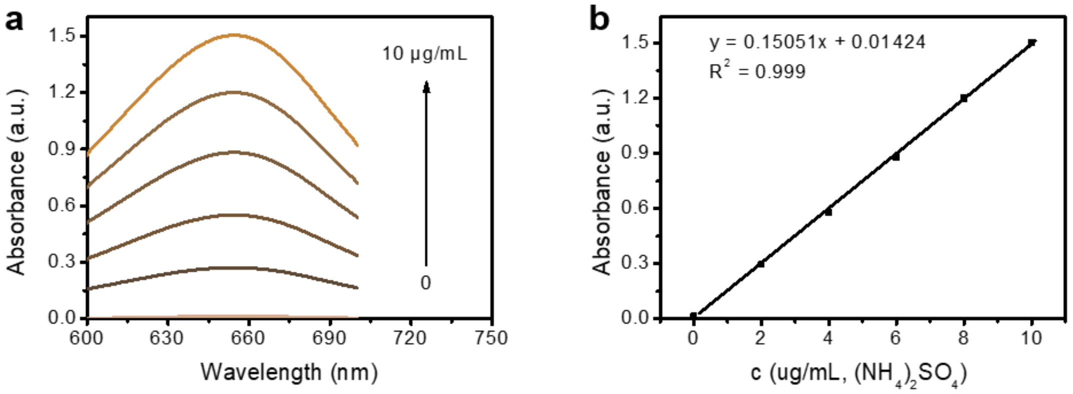

Supplementary Fig. 5. (a) UV-vis adsorption spectra and (b) standard curves for ammonia at pH13.

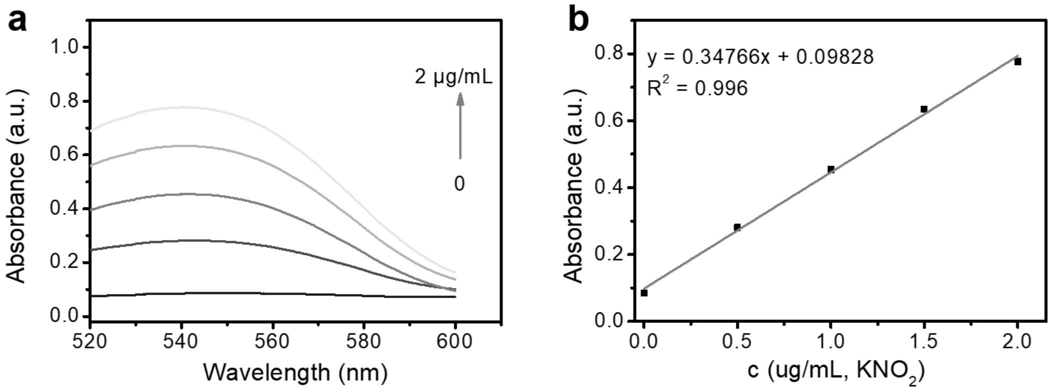

Supplementary Fig. 6. (a) UV-vis adsorption spectra and (b) standard curves for nitrite at pH1.

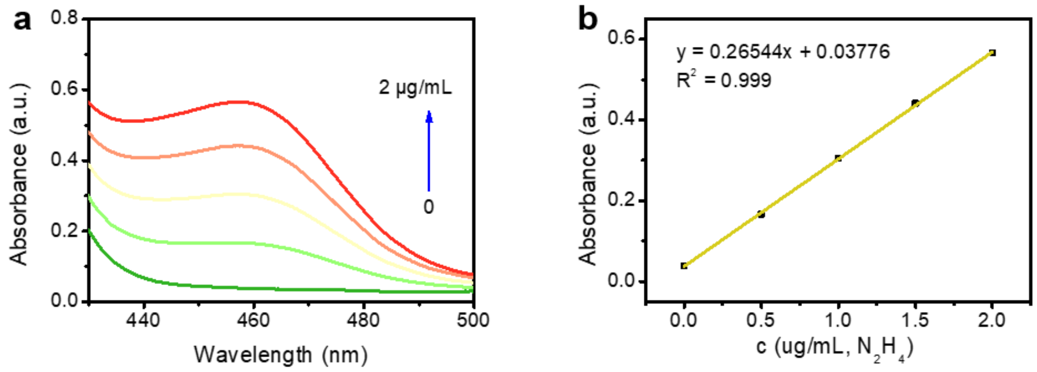

Supplementary Fig. 7. (a) UV-vis adsorption spectra and (b) standard curves for N_2H_4 at pH1.

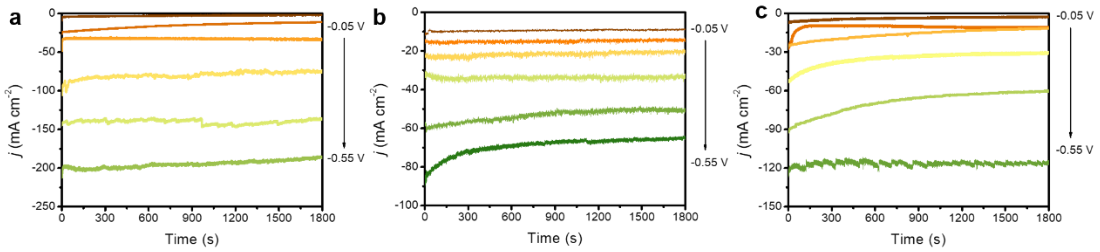

Supplementary Fig. 8. Chronoamperometric curves of TiO_2 nanosheet in (a) pH1, (b) pH7 and (c) pH13 at different potentials.

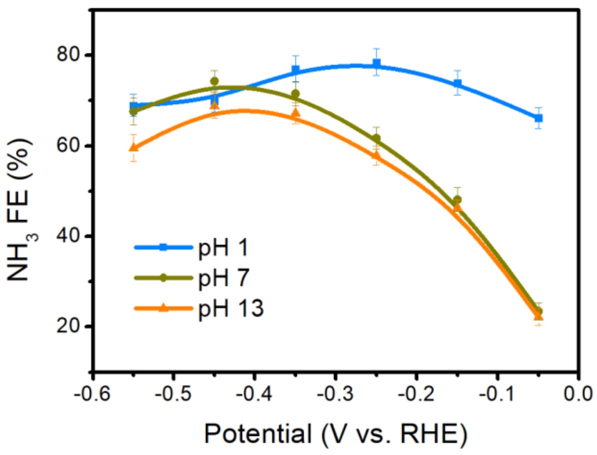

Supplementary Fig. 9. NH_3 FE of TiO_2 at pH1, pH7 and pH13 with 0.5 M NO_3^- in the electrolyte. Error bars are determined from three replicate trials at different potentials.

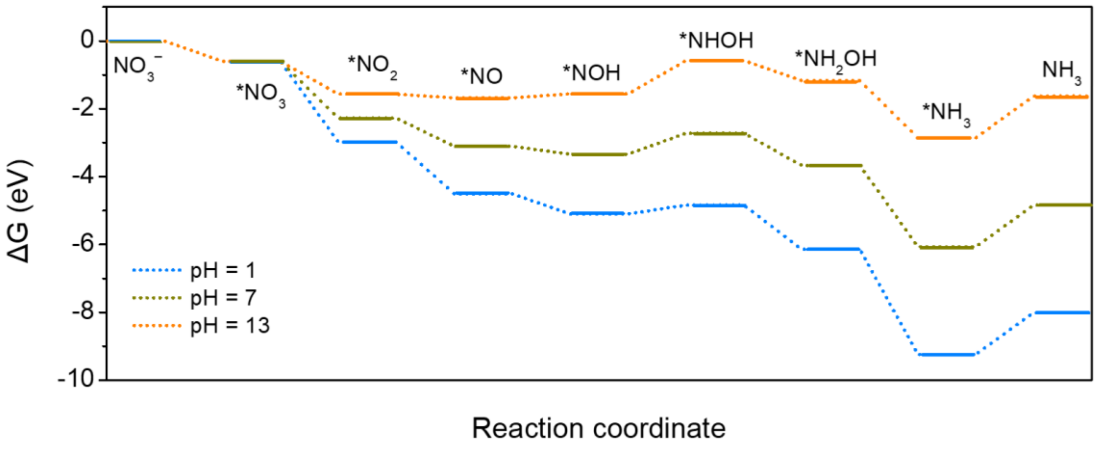

Supplementary Fig. 10. Gibbs free energies for NO_3^-RR on TiO_2 at different pH values of 1, 7 and 13. The pH correction follows the reported literature (ACS Catal. 2021, 11, 23, 14417–14427). As the pH increases from 1 to 7 and 13, a concomitant increase in the free energies for each step of NO_3^-RR is observed due to the sluggish kinetics of H^+ produced from additional water dissociation.

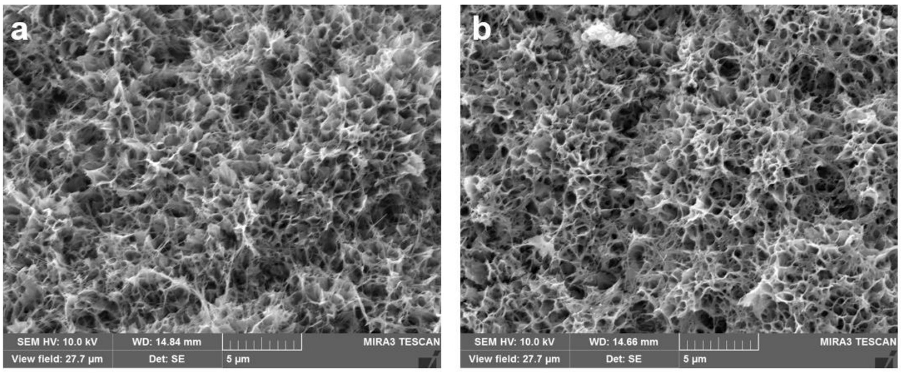

Supplementary Fig. 11. (a) SEM images of (a) TiO_2 and (b) FePc/TiO_2-2.

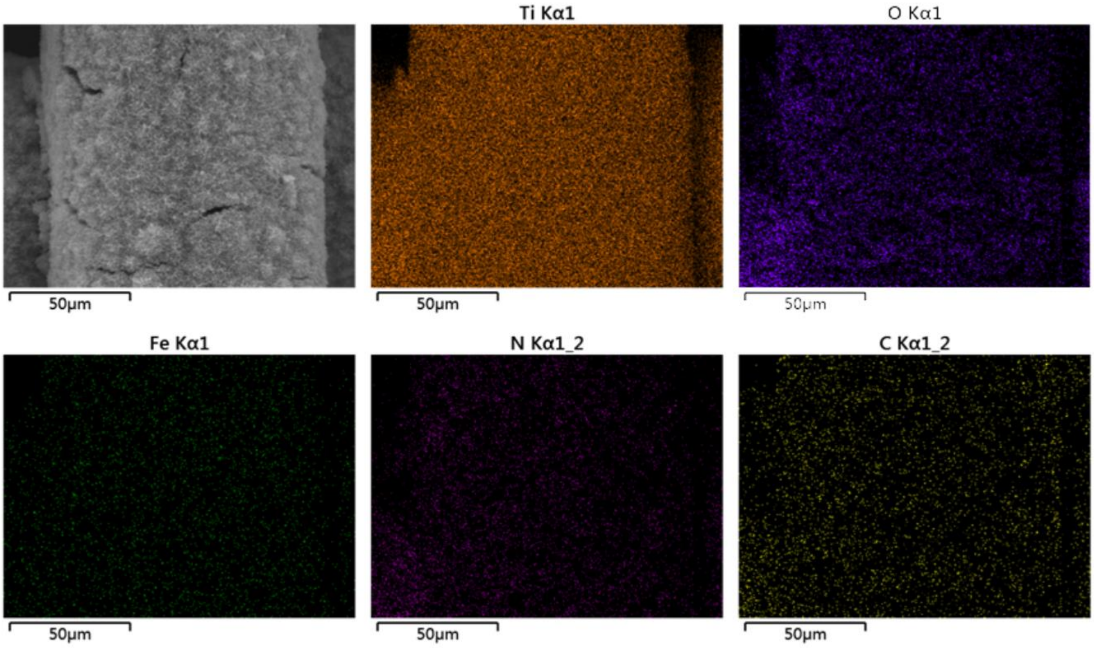

Supplementary Fig. 12. SEM image and mappings of different elements (Ti, O, Fe, N and C) in FePc/TiO_2-2, indicating the uniform distribution of Ti, O, Fe, N and C in FePc/TiO_2-2.

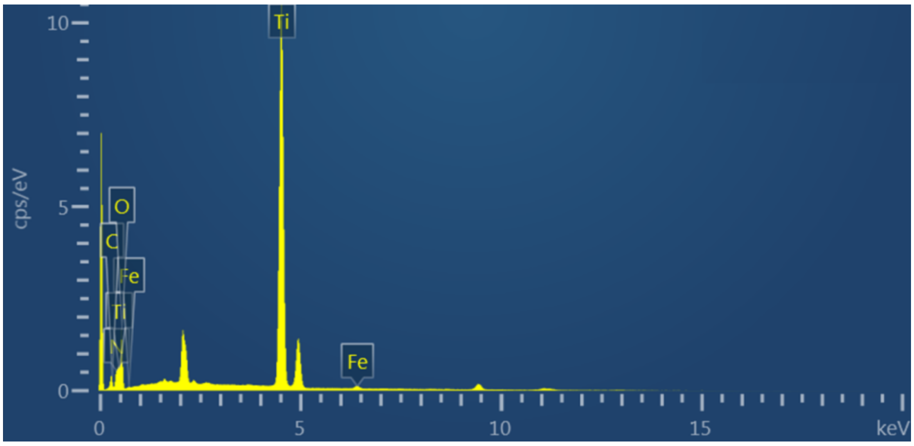

Supplementary Fig. 13. EDS spectrum of FePc/TiO_2-2, which indicates the presence of Ti, O, Fe, N and C in FePc/TiO_2-2.

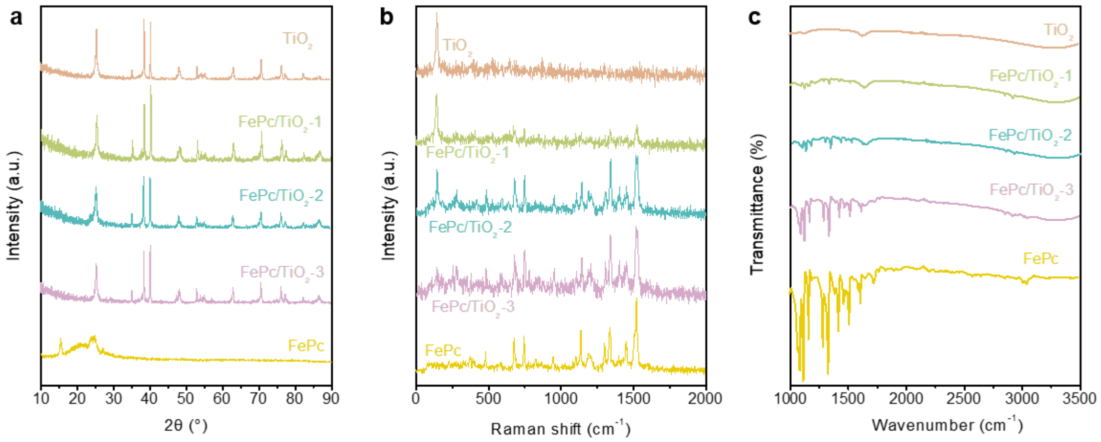

Supplementary Fig. 14. (a) XRD patterns, (b) Raman spectra and (c) FTIR spectra of different samples.

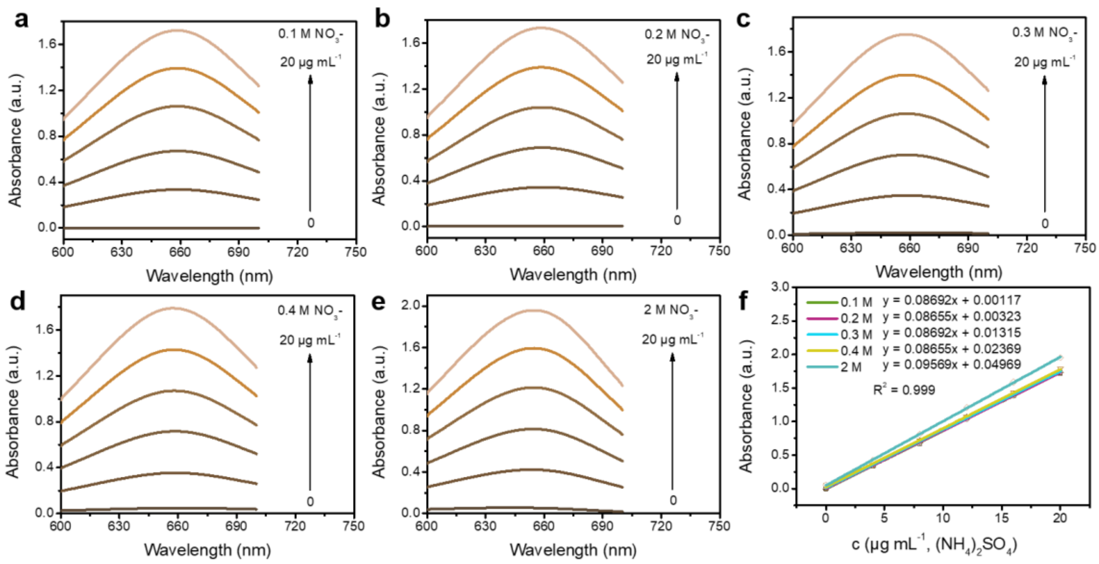

Supplementary Fig. 15. (a-e) UV-vis adsorption spectra for ammonia in different NO_3^- concentrations (pH 1). (f) Corresponding standard curves.

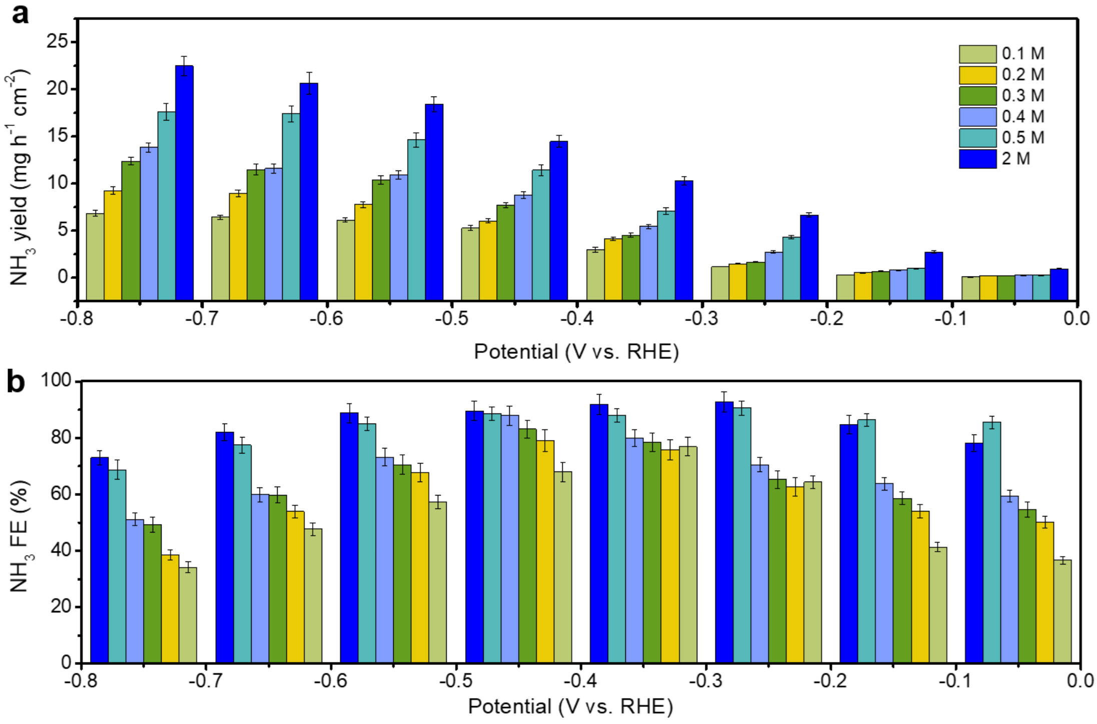

Supplementary Fig. 16. (a) NH_3 yield and (b) NH_3 FE of FePc/TiO_2-2 at different potentials and different NO_3^- concentrations of 0.1 M, 0.2 M, 0.3 M, 0.4 M, 0.5 M and 2 M. Error bars are determined from three replicate trials at different potentials.

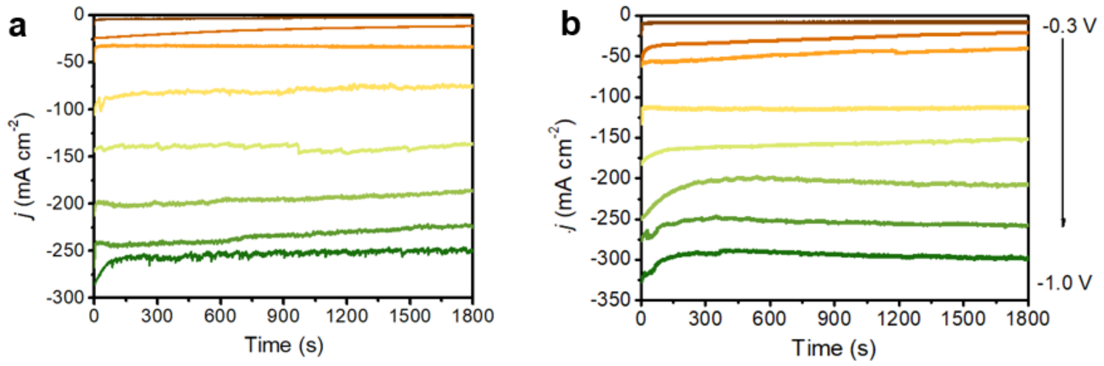

Supplementary Fig. 17. Chronoamperometric curves of (a) TiO_2 and (b) FePc/TiO_2-2 in acid at different potentials.

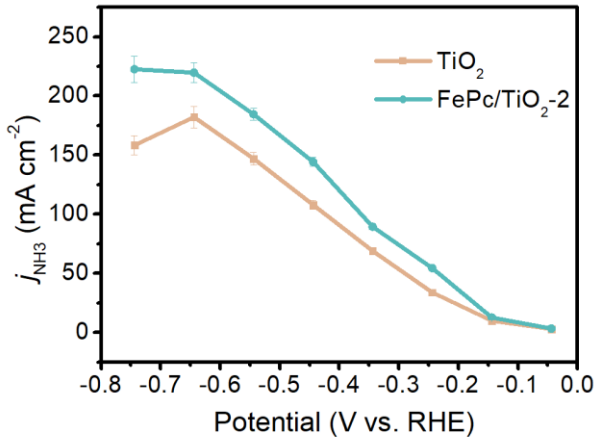

Supplementary Fig. 18. Partial current densities of NH_3 (j_{NH3}) of TiO_2 and FePc/TiO_2-2 for NO_3^-RR in NO_3^- (pH 1). Error bars are determined from three replicate trials at different potentials.

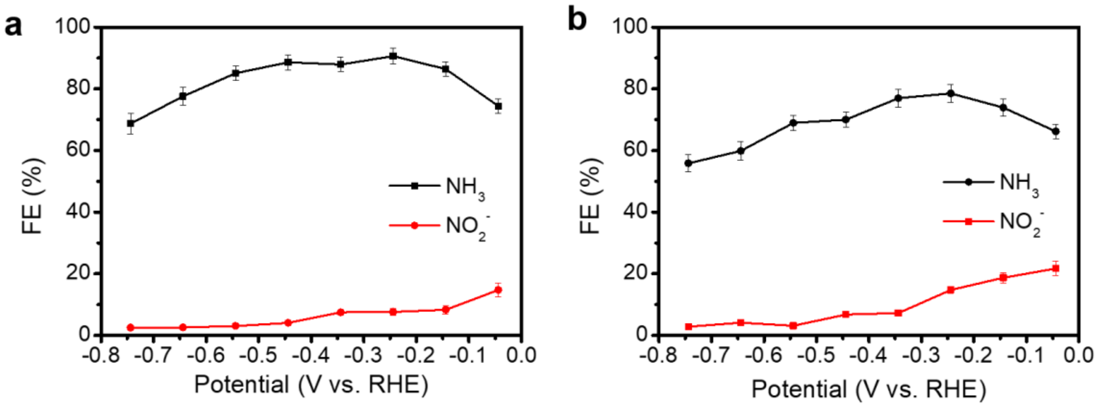

Supplementary Fig. 19. NO_2^- FE of (a) FePc/TiO_2-2 and (b) TiO_2 at pH1 with 0.5 M NO_3^-. Error bars are determined from three replicate trials at different potentials.

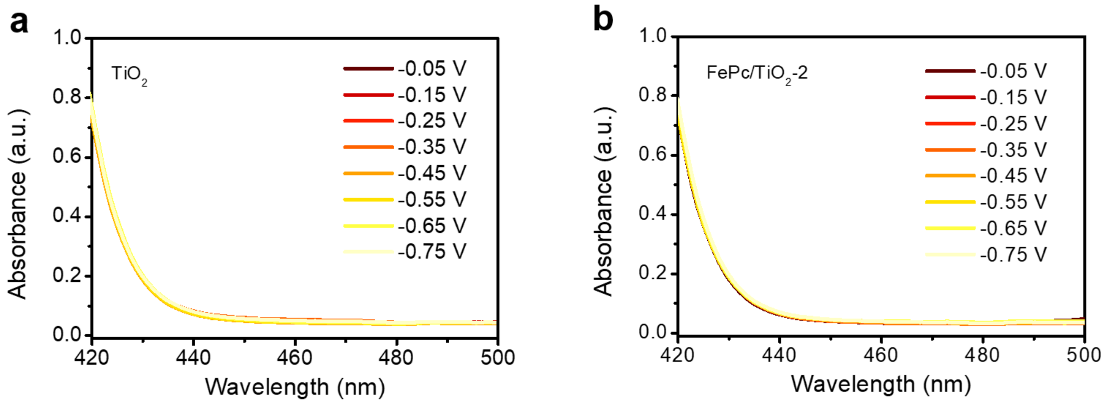

Supplementary Fig. 20. UV-Vis. adsorption curves for (a) FePc/TiO_2-2 and (b) TiO_2 at pH1 with 0.5 M NO_3^- after electrolysis at different potentials for 30 min.

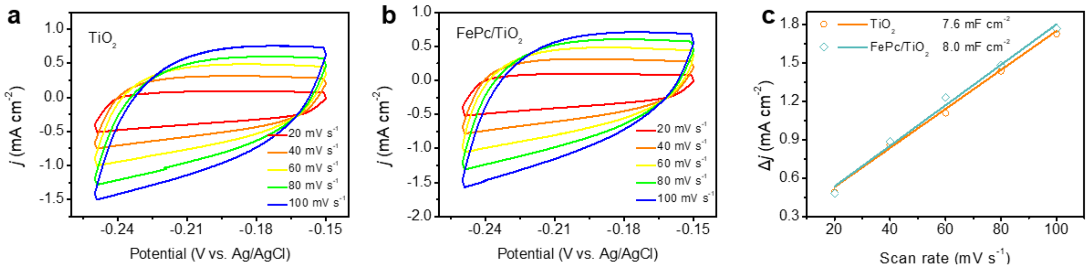

Supplementary Fig. 21. Cyclic voltammetry measurements with various sweep rates (5, 20, 40, 60, 80 and 100 mV s^{-1}) for (a) TiO_2 and (b) FePc/TiO_2-2. (c) Plots of the Δj versus scan rates.

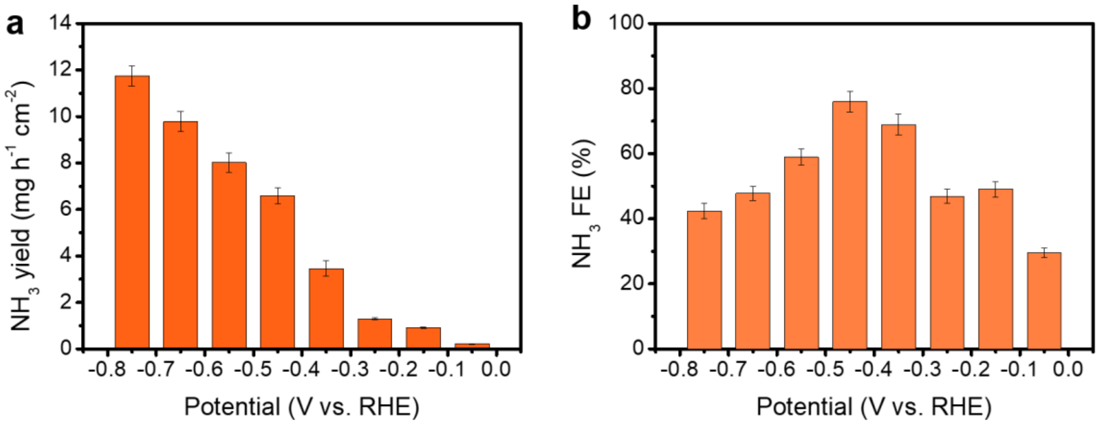

Supplementary Fig. 22. (a) NH_3 yield and (b) NH_3 FE of FePc in 0.5 NO_3^- (pH = 1). Error bars are determined from three replicate trials at different potentials.

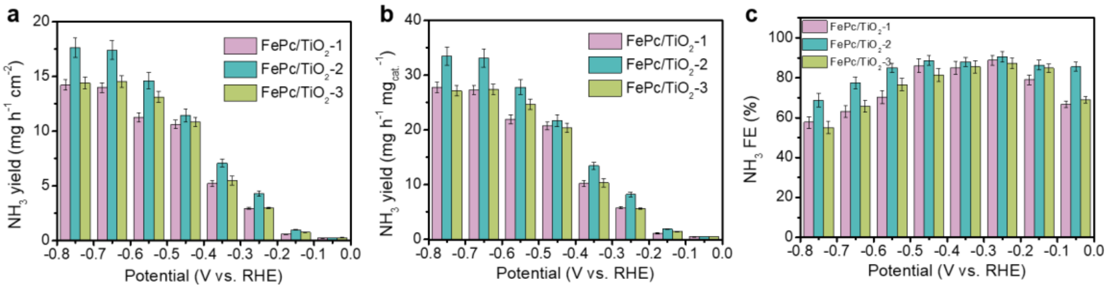

Supplementary Fig. 23. NH_3 yield normalized by (a) geometrical area and (b) catalyst mass and (c) NH_3 FE of FePc/TiO_2-1, FePc/TiO_2-2 and FePc/TiO_2-3 at different potentials 0.5 M NO_3^- (pH 1). Error bars are determined from three replicate trials at different potentials.

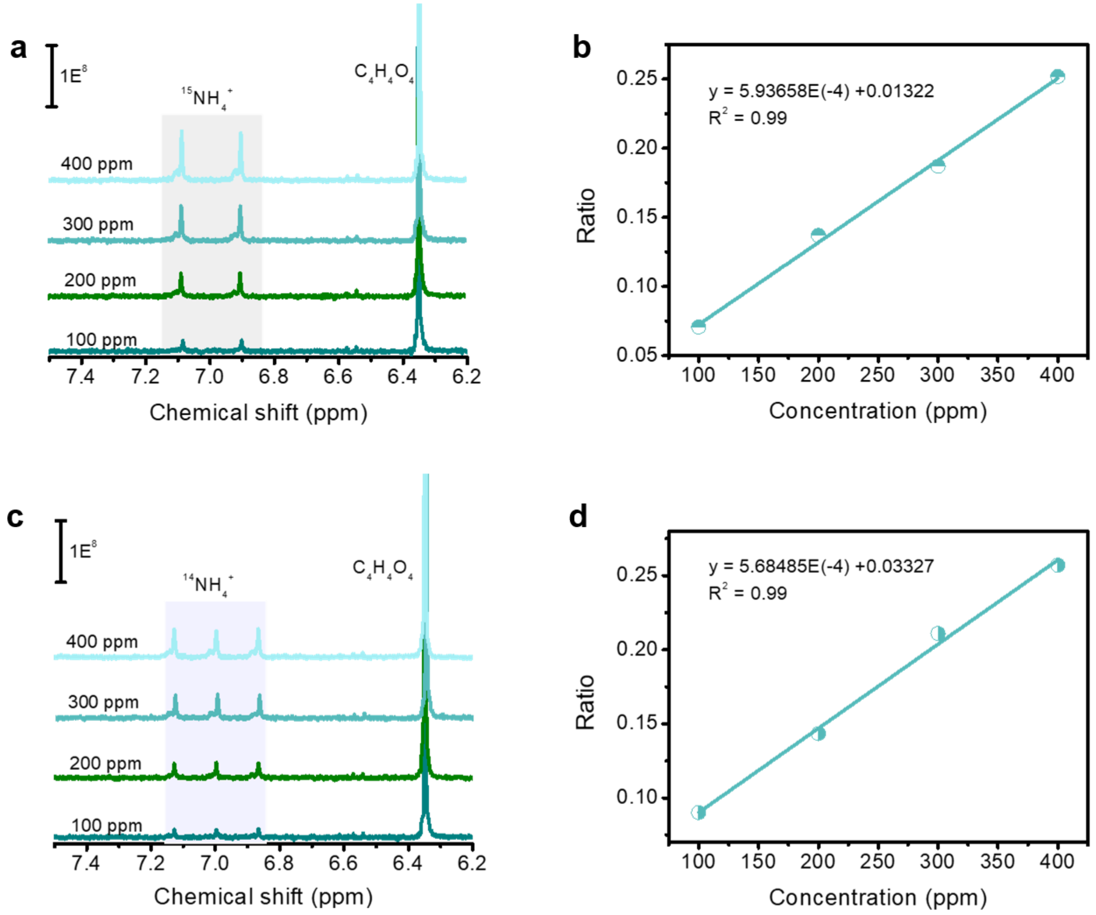

Supplementary Fig. 24. (a) NMR spectra of $(^{15}NH_4)_2SO_4$ standard solution with concentration from 100 ppm to 400 ppm. (b) Established calibration curve for $(^{15}NH_4)_2SO_4$ standard solution. (c) NMR spectra of $(^{14}NH_4)_2SO_4$ standard solution with concentration from 100 ppm to 400 ppm. (d) Established calibration curve for $(^{14}NH_4)_2SO_4$ standard solution.

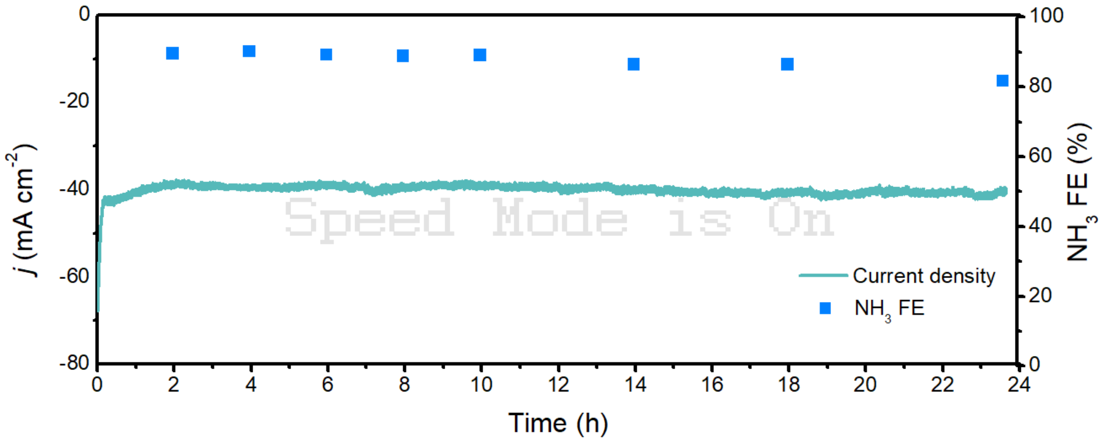

Supplementary Fig. 25. Chronoamperometry curve of FePc/TiO_2-2 at −0.25 V in 0.5 M NO_3^- solution (pH 1) for NO_3^-RR and the NH_3 FE at different times.

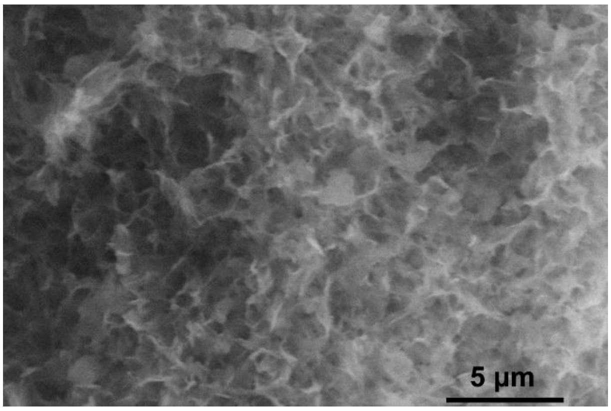

Supplementary Fig. 26. SEM image of FePc/TiO_2-2 after electrolysis at −0.25 V. It should be noted that FePc/TiO_2-2 still keeps nanosheet structure, indicating the good structure stability of FePc/TiO_2-2.

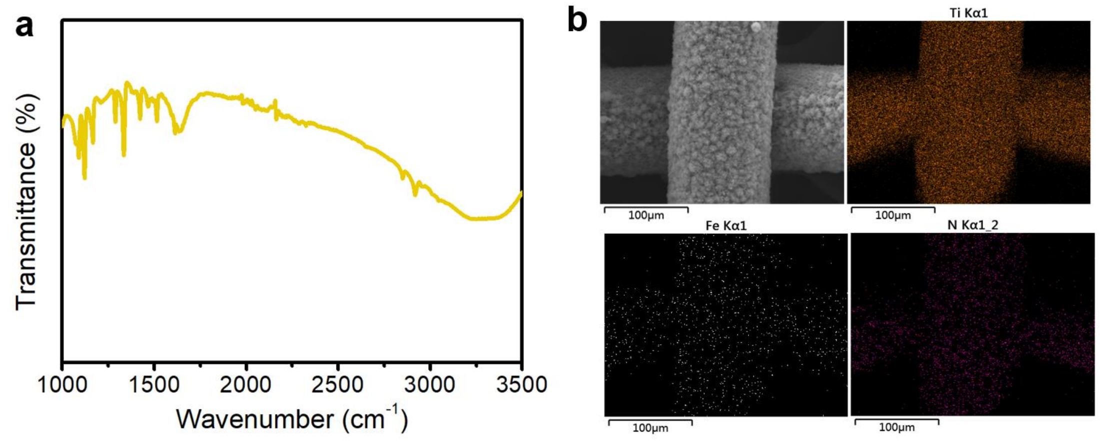

Supplementary Fig. 27. (a) FTIR spectrum and (b) SEM image and corresponding EDS mappings of $FePc/TiO_2$ after stability test.

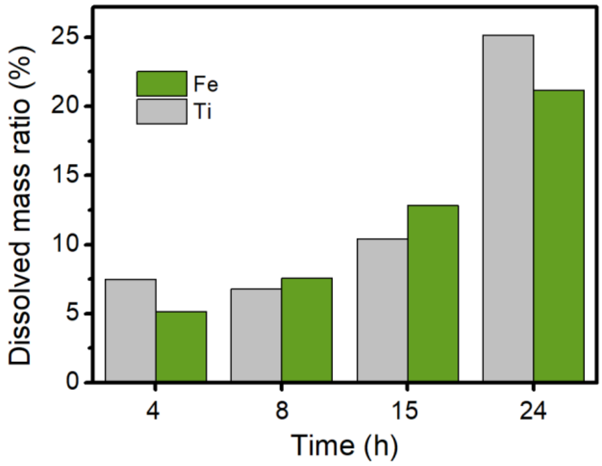

Supplementary Fig. 28. Dissolved mass ratio of Fe and Ti in the solution from the $FePc/TiO_2$-2 catalyst electrode after electrolysis for different time. Only 5.17 wt% of Fe dissolved in the first 4 hours, and this figure increases to about 20 wt% after 24-hour electrolysis. It should be noted that Ti element is also detected in the solution, suggesting the inevitably partial dissolution of the overall $FePc/TiO_2$-2 electrode in the acidic conditions after long-term electrolysis

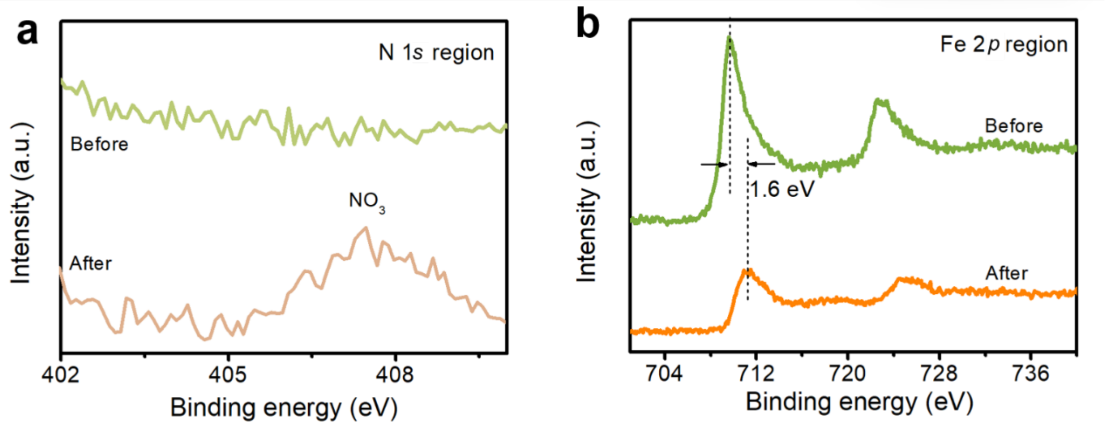

Supplementary Fig. 29. XPS spectra of FePc/TiO_2-2 in the (a) N 1*s* region and (b) Fe 2*p* region before and after immersion in 0.5 M NO_3^-.

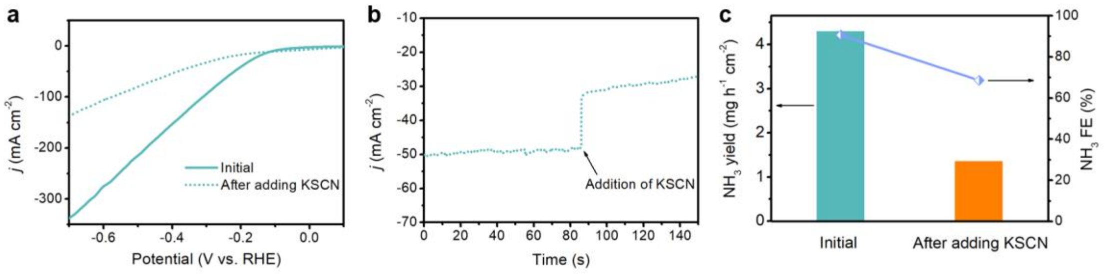

Supplementary Fig. 30. (a) LSV curves of FePc/TiO_2-2 in 0.5 M NO_3^- solution with and without adding 0.02 mM KSCN. (b) Changes in current density before and after KSCN injection in 0.5 M NO_3^- solution at −0.25 V. (c) Comparison in terms of NH_3 yield and NH_3 FE of FePc/TiO_2-2 between initial and after the injection of KSCN.

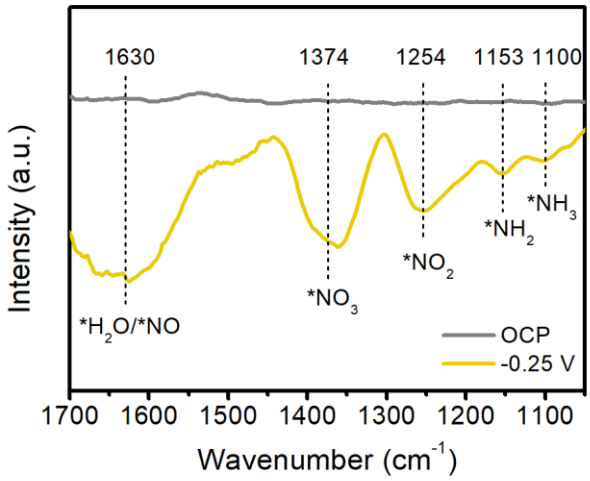

Supplementary Fig. 31. In-situ FTIR spectra of FePc at OCP and −0.25 V in acidic conditions.

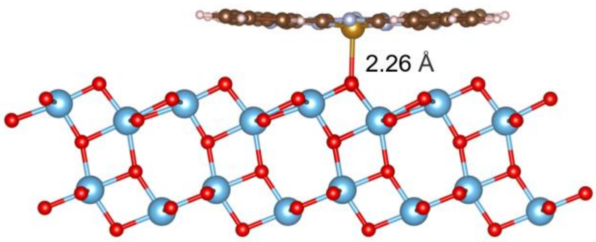

Supplementary Fig. 32. FePc adsorption on TiO_2(101) surface. Blue, red, pink, brown and curry balls represent Ti, O, H, Fe and C atoms, respectively.

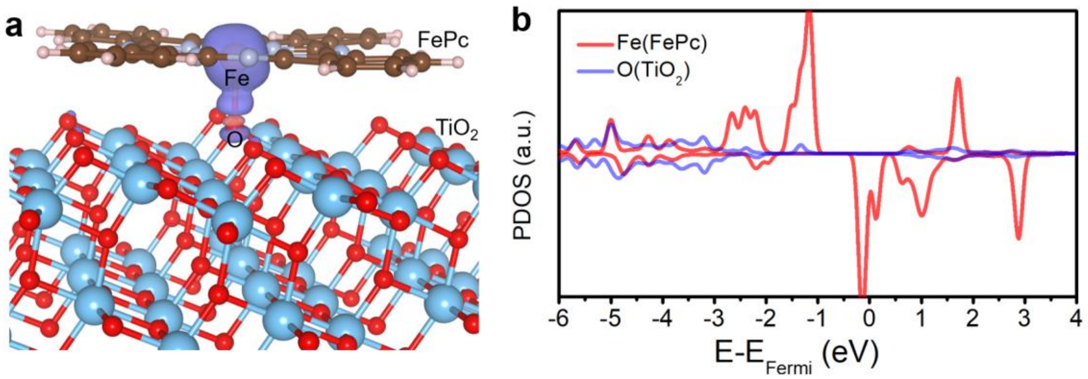

Supplementary Fig. 33. (a) Charge density difference for FePc/TiO_2 system. (b) Density of states of Fe-O orbitals in FePc/TiO_2.

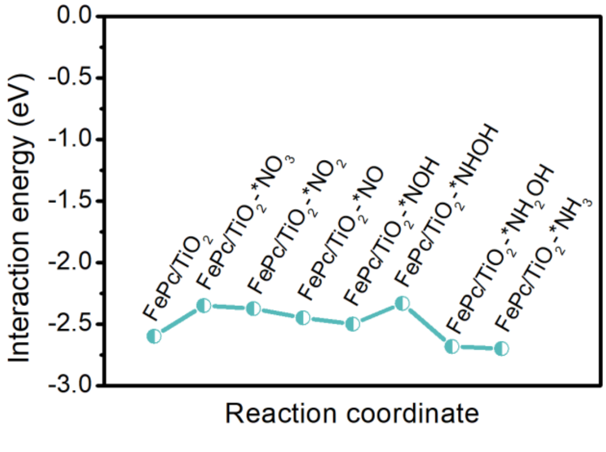

Supplementary Fig. 34. Interaction energy between FePc and TiO_2 in the composite before and after adsorbed with different N-containing intermediates for NO_3^-RR.

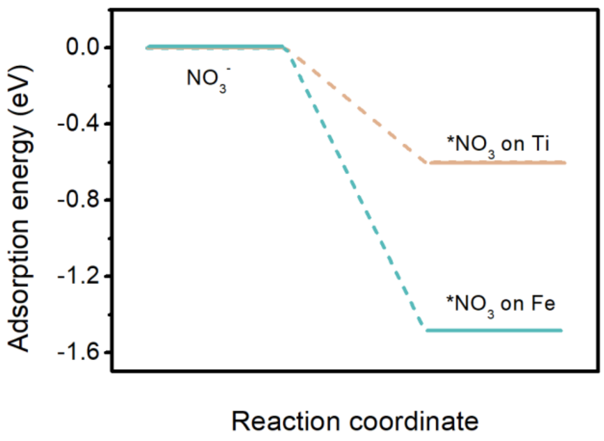

Supplementary Fig. 35. Adsorption energy of NO_3^- at Ti and Fe site of FePc/TiO_2. The negative values indicates that the NO_3^- can be efficiently adsorbed on metal sites. More negative of the adsorption energy of NO_3^- on Fe site indicates stronger adsorption ability of Fe site than that for Ti site.

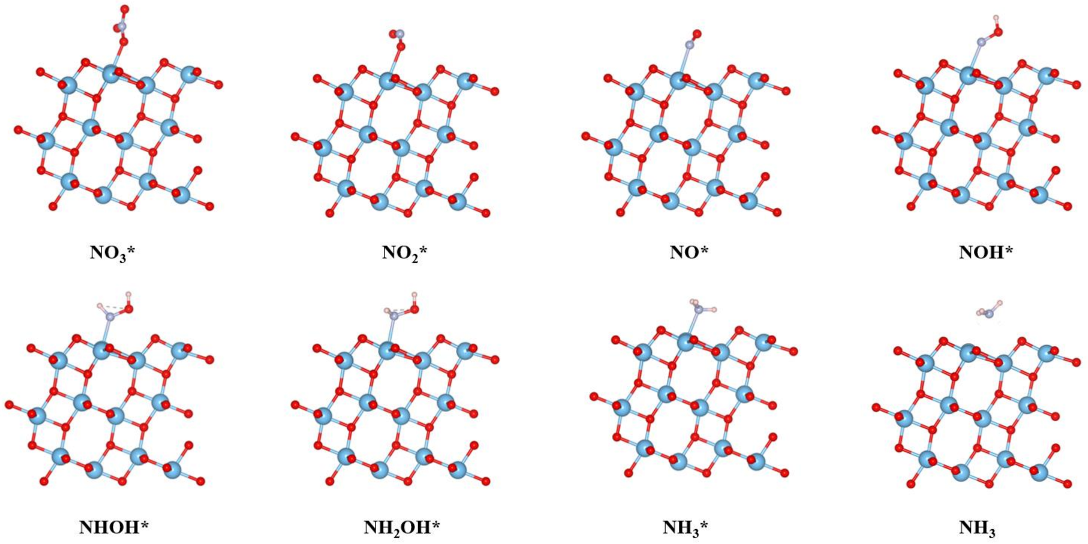

Supplementary Fig. 36. Atomic configuration of N-containing intermediate adsorbed on TiO_2. Blue, red, pink and silver balls represent Ti, O, H and N atoms, respectively.

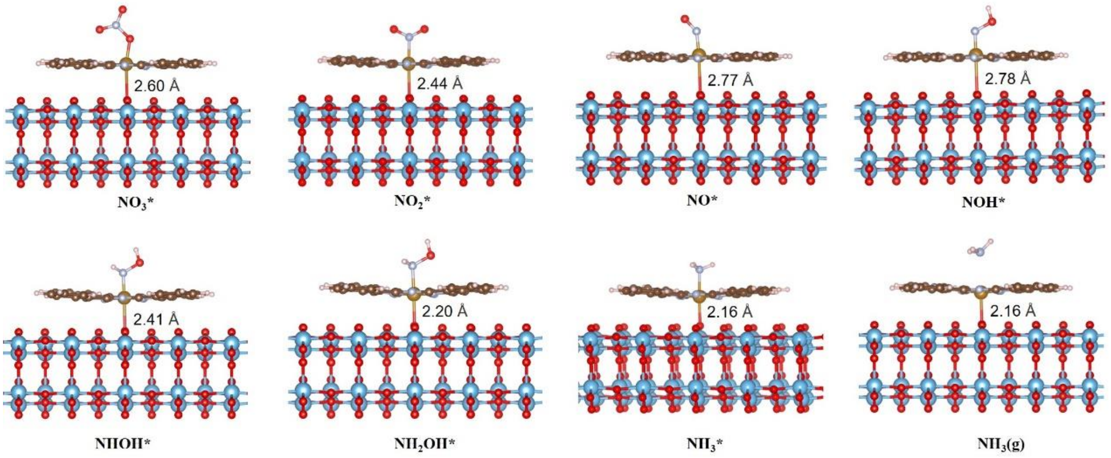

Supplementary Fig. 37. Atomic configuration of N-containing intermediate adsorbed on FePc/TiO_2. Blue, red, pink, brown, curry, silver balls represent Ti, O, H, Fe, C and N atoms, respectively.

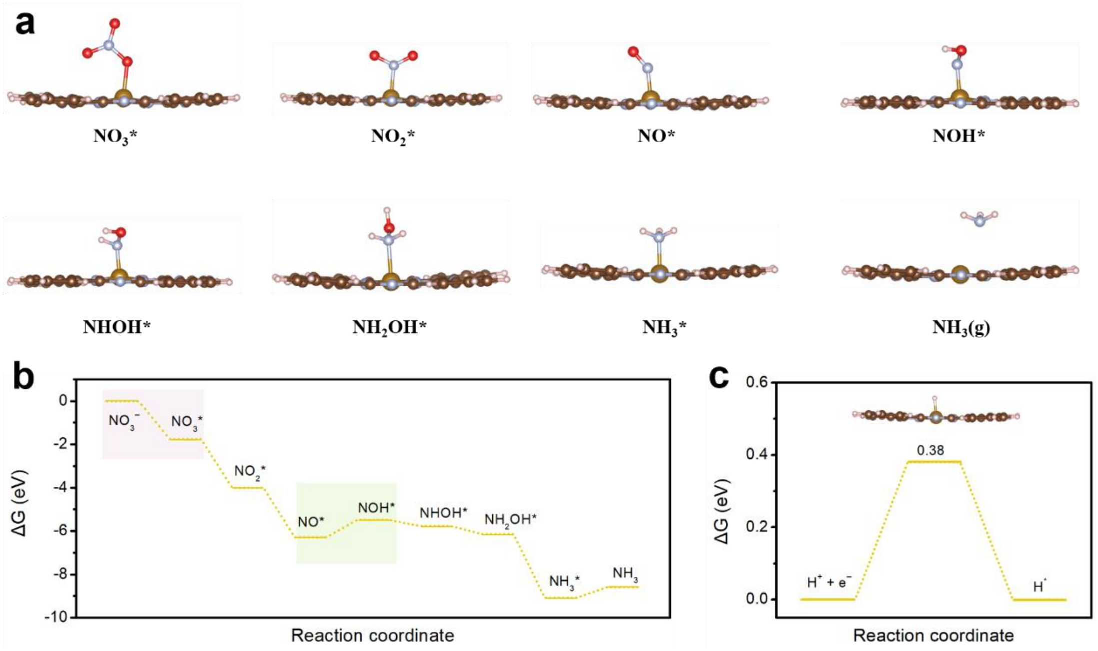

Supplementary Fig. 38. (a) Atomic configurations of N-containing intermediates adsorbed on FePc. Free energy diagram for (b) NO_3^- reduction and (c) HER on the FePc (Inserted is the adsorption configuration of H^* on FePc). Red, pink, brown, curry, silver balls represent O, H, Fe, C and N atoms, respectively.

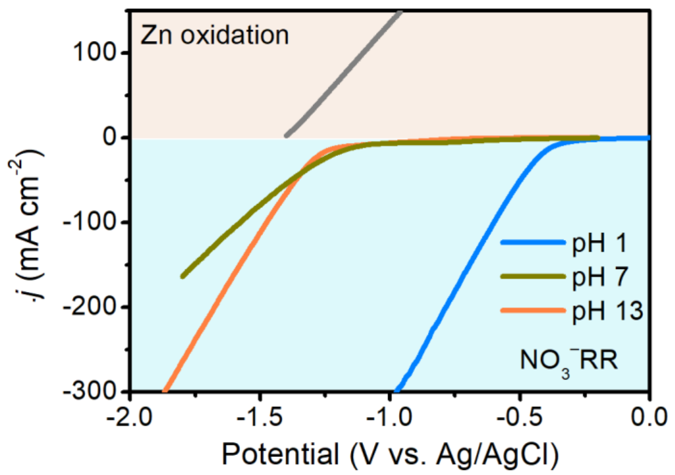

Supplementary Fig. 39. LSV curves of anodic Zn oxidation of Zn plate and the cathodic NO_3^-RR using FePc/TiO_2-2 at different pH values with potentials versus Ag/AgCl reference electrode.

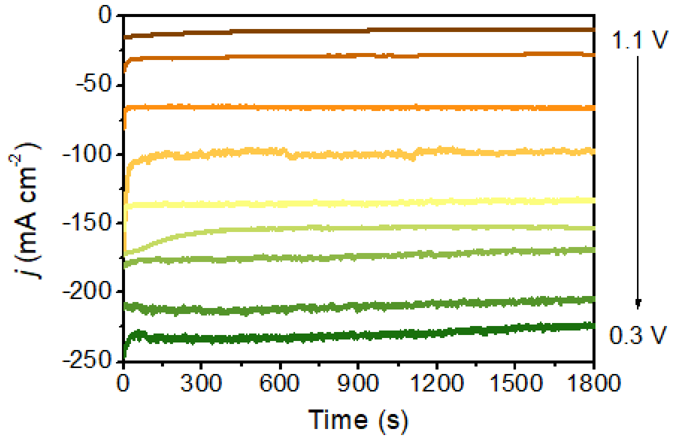

Supplementary Fig. 40. Discharging curves of FePc/TiO_2-based Zn-nitrate battery at different voltages.

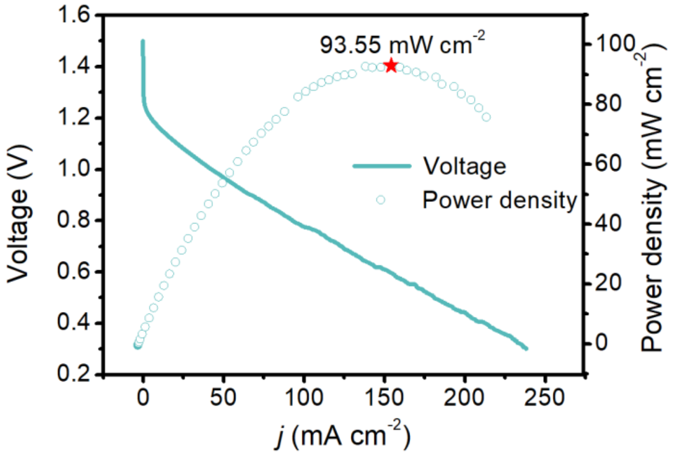

Supplementary Fig. 41. The discharge curve of flow AAHZNB and the corresponding power density.

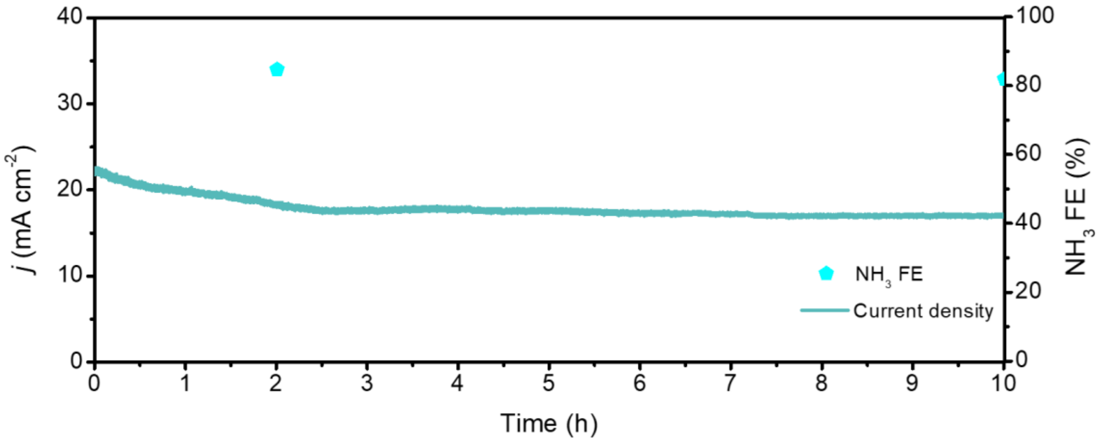

Supplementary Fig. 42. Chronoamperometric curve of our acid-base hybrid battery for 10 hours and the NH_3 FE at different times.

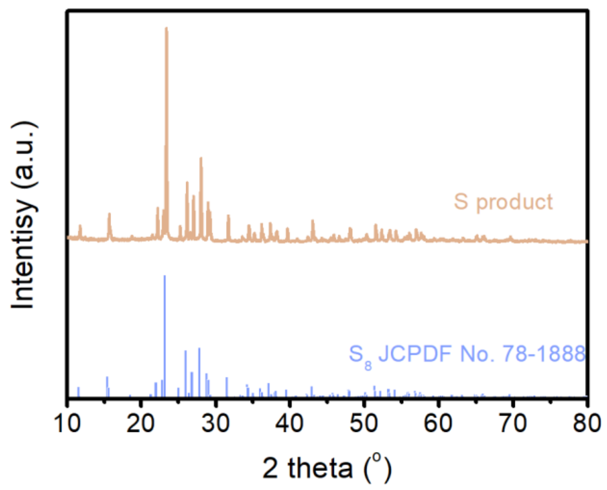

Supplementary Fig. 43. X-ray diffraction pattern for the collected yellow powder. In detail, the concentrated sulfuric acid was added dropwise into the electrolyte solution until adjusting the pH to 1 in the ice bath, and then yellow product was obtained by a centrifugal separating.

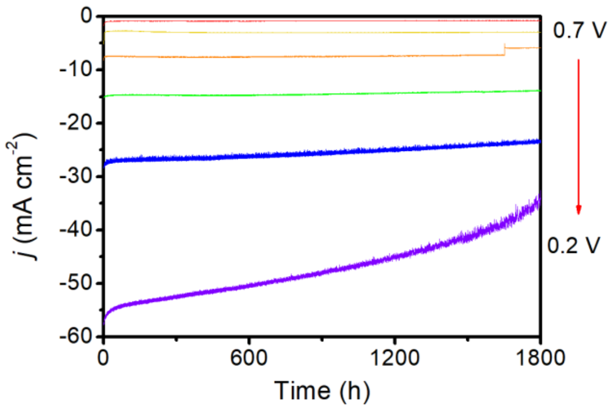

Supplementary Fig. 44. Discharging curves of N_2H_4-NO_3^- fuel cell at different voltages of 0.7 V, 0.6 V, 0.5 V, 0.4 V, 0.3 V and 0.2 V. The discharging current density increases with decreased voltages of N_2H_4-NO_3^- fuel cell.

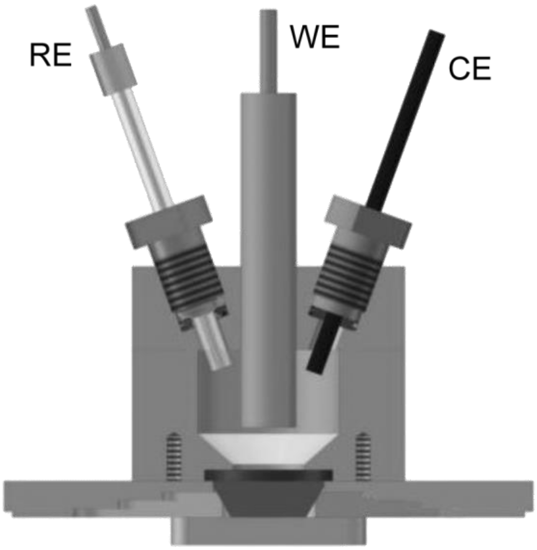

Supplementary Fig. 45. Setup for in-situ electrochemical FTIR test. The catalyst electrode is pressed against the infrared window to form a thin layer of electrolyte (1-10 microns). The infrared light beam passes through the optical window and passes through the thin layer of electrolyte, then reflects the infrared light on the electrode surface, and finally reaches the infrared detector.

Supplementary Table 1. The fitted impendence parameters of TiO_2 at different pH values in 0.5 M NO_3^- solution.

Parameters (Ω)	pH = 1		pH = 7		pH = 13	
	Value	Error (%)	Value	Error (%)	Value	Error (%)
R_1	4.15	0.57	8.86	0.18	3.94	0.10
R_2	52.28	1.06	100.10	0.31	95.04	1.84

Supplementary Table 2. Comparison of the recently reported electrocatalysts for NO_x^- reduction in different electrolytes.

Catalysts	Electrolyte	Maximum NH_3 FE (%)	NH_3 yield (mg h^{-1} cm^{-2})	NH_3 yield (mg h^{-1} $mg_{cat.}^{-2}$)	Ref.
TiO_{2-x}	0.5 M Na_2SO_4/500 ppm NO_3^--N	85	0.3875	0.765	1
Pd/TiO_2	0.25 M $LiNO_3$/5 M LiCl	92.1	1.12	0.112	2
Ru NCs/TiO_2	100 ppm NO_3^-/0.05 M Na_2SO_4	90	10.2	26.8	3
Cu/Fe-TiO_2	50 ppm $NaNO_3$/0.5 M Na_2SO_4	91.2	8.5974	8.5974	4
Co@TiO_2	0.1 M PBS/0.1 M NO_3^-	96.7	13.6	11.33	5
CoP@TiO_2	0.1 M NaOH/0.1 M NO_3^-	96.6	8.4966	-	6
Co_3O_4@TiO_2	0.1 M NaOH/0.1 M NO_3^-	93.1	14.875	9.917	7
Ru_1/TiO_x	1 M KOH/1 M $NaNO_3$	87.3%	9.42	-	8
Ni-TiO_2	0.1 M NaOH/0.1 M NO_2^-	94.89	6.46459	-	9
Ag@TiO_2	0.1 M NaOH/0.1 M NO_2^-	96.4	8.7431	-	10
V-TiO_2	0.1 M NaOH/0.1 M NO_2^-	93.2	9.1936	-	11
Ni@TiO_2	0.1 M NaOH/0.1 M NO_2^-	98.5	9.6679	-	12
NiS_2@TiO_2	0.1 M NaOH/0.1 M NO_2^-	92.1	10.0623	-	13
CuCoSP	0.1 M KOH/0.1 M NO_3^-	93.3	19.89	-	14
Ru nanocluster	1 M KOH/1 M KNO_3	100	19.89	94.52	15
Fe SAC	0.5 M KNO_3/0.1 M K_2SO_4.	75	7.82	20	16
Fe-PPy SACs	0.1 M KOH/0.1 M NO_3^-	100	2.75	11.45	17
Fe-N/P-C	0.1 M KOH/0.1 M NO_3^-	90.3	8.99	17.98	18
Fe-cyano-R NSs	1 M KOH/0.1 M KNO_3	90.4	21.1	42.1	19
CoO_x	0.1 M KOH/0.1 M KNO_3	93.4	6.592	82.4	20
BiOCl	1 M KOH/0.5 M KNO_3	90.6	23.715	46.5	21
Ru-CuW	1 M KOH/0.1 M NO_3^-	95.6	76.512	191.28	22
CuNi alloys	1 M NaOH/44.3 g L^{-1} NO_3^-	97.03	94.57	-	23
0.6W-O-CoP	1 M KOH/0.1 M NO_3^-	80.92	88.9	31.6	24
CoP NAs	1 M KOH/1 M NO_3^-	100	16.25	9.67	25
RuFe NFs	0.1 $NaNO_3$/0.5 M Na_2SO_4.	85.1	7.74	38.68	26
Ru/β-$Co(OH)_2$	1 M KOH/1 M KNO_3	98.78	39.1	24.12	27
Fe_2M-MOF	0.05 M H_2SO_4/50 g L^{-1} KNO_3	90.55	10.65	20.65	28
PA-RhCu	0.1 M $HClO_4$/0.05M NO_3^-	93.7	0.24	2.4	29
**FePc/TiO_2-2**	**0.1 M HNO_3/0.4 M KNO_3**	**90.6**	**17.4**	**33.08**	**This work**

Supplementary Table 3 Comparison between alkaline-acid hybrid Zn-NO_3^- battery and other reported Zn-NO_3^- batteries.

Catholyte	Anolyte	Cathode	OCV (V)	Membrane	NH_3 yield (mg h^{-1} cm^{-2})	P (mW cm^{-2})	Duration (h)	Ref.
**0.1 M HNO_3 + 0.4 M KNO_3**	**6 M KOH**	**FePc/TiO_2-2**	**1.99**	**Bipolar**	**12.3**	**91.4**	10	**This work**
3.5 M NaOH + 44.3 g L^{-1} NO_3^-	3.5 M NaOH	CuNi NPs/CF	0.94	Bipolar	18.1	70.7	12	23
1 M KOH + 0.1 M $NaNO_3$	1 M KOH	W-O-CoP	0.7	Alkaline membrane (FAB-PK-130)	2.79	9.27	5	24
0.5 M Na_2SO_4 + 0.1 M $NaNO_3$	1 M KOH + 0.02 M $Zn(Ac)_2$	RuFe NF	1.37	Bipolar	-	9.5	8	26
1 M KOH + 0.1 M KNO_3	6 M KOH	Ru/β-$Co(OH)_2$	1.48	Nafion 212	6.46	29.87	30	27
0.25 M $LiNO_3$ + 5 M LiCl	5 M KOH	Pd/TiO_2	0.81	Bipolar	0.54	0.87	12	30
0.2 M K_2SO_4 + 0.05 M KNO_3	1 M KOH	Fe/Ni_2P	1.22	Bipolar	0.38	3.25	6	31
1 M KOH + 0.05 M NO_3^-	5 M KOH	MP-Cu	1.27	Nafion 117	1.292	7.56	2.5	32
0.5 M K_2SO_4 + 200 ppm NO_3^-	-	Ni_1Cu-SAA	1.51	Nafion 117	2.1	12.7	5	33
1 M KOH + 1 M KNO_3	1 M KOH + 0.02 M $Zn(Ac)_2$	DM-Co	0.7	Anion membrane	2.04	25.8	32	34
1 M KOH + 1 M $NaNO_3$	1 M KOH	Ru-25CV/NF	1.2	Bipolar	2.9	51.5	3	35
0.1 M NaOH + 0.1 M NO_3^-	6 M KOH	$NiCo_2O_4$	1.3	Nafion 117	0.82	3.94	2.5	36
0.1 M PBS + 0.1 M NO_3^-	1 M KOH	$CoAl_2O_4$	1.862	Bipolar	0.75	3.43	8	37
0.1 M NaOH + 0.1 M NO_3^-	-	$ZnCo_2O_4$	1.52	Nafion 117	1.56	4.62	8	38
3 M KOH + 0.5 M NO_3^-	3 M KOH	Cu nanowire	0.943	Nafion 1110	2.125	14.1	10	39
0.1 M NaOH + 0.1 M NO_3^-	6 M KOH	Ir SAC Co_3O_4	1.396	Nafion 117	0.7633	5.6	2.5	40
-	0.1 M $NaNO_3$	Fe_2TiO_5	1.5	Nafion 117	0.7803	5.6	10	41
1 M KOH + 0.1 M NO_3^-	6 M KOH	NiCoBDC@HsGDY	1.47	Nafion 117	1.125	3.66	3	42
3 M KOH + 0.5 M NO_3^-	3 M KOH + 0.1M $Zn(Ac)_2$	NiRu ball-flower	1.39	Nafion 1110	-	10	~90	43

Supplementary Table 4 Comparison between alkaline-acid hybrid Zn-NO_3^- battery and the reported fuel cells.

Catholyte	Anolyte	Cathode	Anode	P (mW cm^{-2})	Ref.
6 M KOH + 0.2 M $Zn(Ac)_2$		$Co_2N_{0.67}/CoMoO_4$	Zn	20	44
0.5 M H_2SO_4	1 M KOH	CoP/C/Cu	Zn	31	45
0.5 M H_2SO_4	1 M KOH	Mo-WC@NCS	Zn	41.4	46
0.5 M H_2SO_4	4 M NaOH	Pt/CNTs	Zn	80	47
0.2 M K_2SO_4 + 0.05 M KNO_2	1 M KOH	C/Co_3O_4	Zn	6.03	48
18 M KOH + 0.02 M $Zn(Ac)_2$		CoNCNTF/CN	Zn	63	49
11.25 M KOH + 0.25 M ZnO		Co-NC@Al_2O_3	Zn	72.4	50
11.25 M KOH + 0.25 M ZnO		NC-Co_3O_4-90	Zn	82	51
1.25 M KOH + 0.25 M ZnO		Co-FeCo/N-G	Zn	82	52
KOH + $Zn(Ac)_2$		Meso-CoNC@G	Zn	85.6	53
4.2 M NaOH		CoPOF@CNT	Zn	89	54
3 M H_2SO_4	3 M KOH + 6 M urea	Ni-Fe LDH/EG	Pt/C	12	55
Humidified air	3 M KOH + 1 M urea	NiCo 25/MWCNT AG	Pt/C	17.5	56
Humidified air	0.1 M KOH + 50 mM urea	$NiCo_2O_4$	Pt/C	18	57
0.5 M H_2SO_4 + 2 M H_2O_2	2 M KOH + 0.33 M urea	Co dendrites	Pt/C	21	58
0.5 M H_2SO_4 + 2 M H_2O_2	3 M KOH + 0.7 M urea	Ni–Se	Prussian blue	33	59
Humidified air	3 M KOH + 0.7 M urea	NiCu/ZnO@MWCNT	Pt/C	44.36	60
1.0 M KOH + 0.5 M N_2H_4 seawater		FeNiP-NPHC		31	61
1.0 M KOH + 0.5 M N_2H_4		$CoPt_3$/CoPt		38	62
1.0 M KOH	1.0 M KOH + 0.5 M N_2H_4	PW-Co_3N/NF	Pt/C	46.3	63
1.0 M KOH	1.0 M KOH + 0.5 M N_2H_4	Ni3N-Co_3N PNAs/NF	Pt/C	60.3	64
1.0 M KOH	1.0 M KOH + 0.5 M N_2H_4	RP-CPM	Pt/C	64.77	65
1.0 M H_2SO_4	1.0 M H_2SO_4 + 1.0 M HCOOH	Pd-nanoarray@CP	Pt/C	35.8	66
Humidified air	3 M HCOOH	Pt_1Cu_1 NW	Pt/C	63.7	67
		Pt_5Cu_1 NW		75	
**0.1 M HNO_3 + 0.4 M KNO_3**	**6 M KOH**	**FePc/TiO_2-2**	**Zn plate**	**91.4**	**This work**

Supplementary References

1. ACS Catal. 2020, 10, 3533.
2. Energy Environ. Sci. 2021, 14, 3938.
3. Small 2023, 2300437.
4. Appl. Catal. B 2023, 325, 122360.
5. Small, 2023, 2208036.
6. Mater. Today Phys. 2022, 28, 100854.
7. J. Colloid Interface Sci. 2023, 630, 714.
8. Angew. Chem.Int. Ed.2022,61,e2022082.
9. Mater. Today Energy, 2023, 31, 101220.
10. Inorg. Chem. Front., 2023, 10, 1431.
11. Materials Today Phys. 2023, 30, 100944.
12. ACS Sustainable Chem. Eng. 2023, 11, 2686.
13. J. Colloid Interface Sci. 2023, 634, 86.
14. Nat. Commun. 2022, 13, 1129.
15. J. Am. Chem. Soc. 2020, 142, 7036.
16. Nat. Commun. 2021, 12, 2870.
17. Energy Environ. Sci. 2021,14, 3522.
18. Angew. Chem. Int. Ed. DOI:10.1002/anie.202308044
19. ACS Nano 2022, 16, 1072.
20. ACS Catal. 2021, 11, 15135.
21. ACS Nano 2022, 16, 4795.
22. Nat. Nanotechnol. 2022, 17, 759.
23. Energy Environ. Sci. 2023, 16, 2991.
24. Adv. Mater. 2023, 2304508.
25. Energy Environ. Sci. 2022, 15, 760.
26. Proc. Natl. Acad. Sci. U.S.A. 2023, 120, e2306461120.
27. Energy Environ. Sci., 2023,16, 2483.
28. Angew. Chem. Int. Ed. 2023, 62 202305246.
29. Adv. Energy Mater. 2022, 12, 2103916.
30. Energy Environ. Sci. 2021, 14, 3938.
31. Adv. Energy Mater. 2022, 12, 2103872.
32. Adv. Funct. Mater. 2022, 32, 2212236.
33. Appl. Catal. B 2022, 316, 121683.
34. Adv. Funct. Mater. 2022, 32, 2209464.
35. Small 2022, 12, 2200436.
36. Small 2022, 18, 2106961.
37. Chem. Eng. J. 2022, 435, 135104.
38. Mater. Today Phys. 2022, 23, 100619.
39. Angew. Chem. Inter. Ed. 2023, 62, e202218717.
40. Chem. Catal. 2023, 3, 100477.
41. Angew. Chem. Inter. Ed. 2023, 135, e202215782.
42. ACS Nano 2023, 17, 7, 6687.
43. Angew. Chem. Int. Ed. 2023, 135, e202305695.
44. Chem. Eng. J. 2022, 435, 134795.
45. Chem. Eng. J. 2022, 448, 137716.
46. Nano Energy 2020, 74, 104850.
47. Angew. Chem. 2018, 130, 3974.
48. Energy Environ. Sci. 2022, 15, 3024-3032.
49. Carbon 2019, 142, 379.

50. Adv. Mater. 2018, 30, 1805268.
51. Adv. Mater. 2017, 29, 1704117.
52. Appl. Catal. B 2019, 256, 117887.
53. Adv. Mater. 2017, 29, 1704898.
54. ACS Energy Lett. 2021, 6, 2491.
55. ChemSusChem 2022, 15, e202102614.
56. Sci. Rep. 2019, 9, 479.
57. J. Mater. Chem. A 2018, 6, 23019.
58. Appl. Surf. Sci. 2021, 555, 149698.
59. Sustain. Energy Rev. 2021, 150, 111470.
60. Electrochim. Acta 2018, 261, 78.
61. Adv. Funct. Mater. 2022, 32, 2205767.
62. eScience 2022, 2, 416.
63. Nat. Commun. 2020, 11, 1853.
64. Angew. Chem., Int. Ed. 2021, 60, 5984.
65. Sci. Adv. 2020, 6, eabb4197.
66. Adv. Funct. Mater. 2022, 32, 2201872.
67. ACS Appl. Mater. Inter. 2022, 14, 11457.
